# Exploring Food Security, Functional Limitations, and Quality of Life Among Adults 60 Years and Older in New York City: A Cross‐Sectional Study

**DOI:** 10.1155/jare/1291602

**Published:** 2026-05-25

**Authors:** Adjara Stewart, Christine Rodriguez, Destiny Lopez, Nelia Jose, Mya Encarnacion, Sheila Hankin, Elgloria A. Harrison, Apeksha Mewani, Collette M. Brown

**Affiliations:** ^1^ Health, Administration, Research and Technology, School of Health Sciences, Human Services and Nursing, Lehman College, Bronx, New York, 10468, USA; ^2^ Department of Nursing, School of Health Sciences, Human Services, and Nursing, Lehman College, Bronx, 10468, New York, USA

## Abstract

**Background and Purpose:**

Food insecurity and activity limitations impact the overall health‐related quality of life (HRQOL) of older adults. This cross‐sectional study investigates the relationship between food security and functional limitation on the QOL life among older adults in New York City.

**Methodology:**

A total of 378 participants aged 60 years and older were assessed using the USDA 6‐Item Household Food Security Survey, and the Eastern Cooperative Oncology Group (ECOG) scale was used to assess overall functional status and activity limitation and the CDC’s HRQOL‐4 questionnaires.

**Results:**

Results indicated that 19.1% of the participants were food insecure, 56.9% reported fully active, and 33.9% reported very good or excellent overall health. Participants who experienced food insecurity were more likely to rate their overall health as poor or fair (*p* = 0.031) and report 12 or more days that their physical or mental health kept them from doing their usual activities (*p* = 0.001) compared to those who were food secure. Similarly, older adults with more functional limitations or disabilities were strongly associated with poorer HRQOL (*p* < 0.001) in all domains of the HRQOL‐4.

**Conclusion:**

These results show the impact of food insecurity and functional limitations and HRQOL in older adults. The results emphasize the urgent need for addressing food insecurity and promote healthy aging among older adults in New York City.

## 1. Introduction

In 2022, the United Statesolder adult population was 57.8 million, a 34% increase since 2012. It is estimated that, by 2030, older adults (65 years and older) will represent 21% of the American population [1]. In New York City, the aging population faces difficulties that impact their health and general well‐being. This growing demographic trend has significant implications for public health, particularly in urban settings where older adults face unique functional challenges that affect quality of life (QOL).

Food insecurity is a socioeconomic condition of limited or uncertain access to adequate and safe foods to sustain their daily lives [2, 3], and among older adults, it is strongly associated with poorer health‐related QOL (HRQOL). The prevalence of food insecurity among adults aged 60 and older in New York State is approximately 10% [4]. Studies show that food‐insecure older individuals are more likely to report increased psychological distress, limited physical functioning, and a higher number of unhealthy days [5–8]. In New York City, these effects are intensified by the high cost of living, social isolation, and barriers to accessing services [6]. A recent study by Brown et al. [8] found that food insecurity was linked to greater physical activity limitations and more days of poor health. Similarly, Muñoz‐López et al. [9] reported that older adults consuming only one full meal per day had significantly lower QOL scores (*p* < 0.001). These vulnerabilities are associated with increased emotional distress, limited independence, and the onset or worsening of chronic conditions [5, 8, 10]. As the aging population continues to grow, these compounded burdens pose serious public health challenges requiring targeted interventions.

Recent research has shown that functional impairments are not merely physical limitations but serve as early indicators of broader functional decline and poor health outcomes [11], which are linked to higher hospitalization rates, increased caregiving needs, mortality risk, and diminished QOL [11, 12]. Functional limitations in ADLs such as bathing, dressing, and mobility are powerful indicators of declining health and diminished autonomy in aging populations. Moreover, older adults experiencing both ADL and instrumental activities of daily living (IADL) limitations are more likely to report emotional distress, social withdrawal, and depressive symptoms, which further impact emotional resilience and day‐to‐day independence [13]. In urban settings like New York City, where older adults may face added mobility challenges and environmental barriers, the impact of ADL limitations may be even more profound. Functional impairments not only reduce independence but are also consistently associated with emotional distress and depressive symptoms in older adults, compounding the burden of aging and illness [14–16]. These findings highlight the urgent need to examine how ADL impairments relate to multiple dimensions of QOL in urban older adult populations.

When food insecurity and functional limitations intersect, the burden on older adults’ QOL may be compounded. While each factor independently poses challenges, their co‐occurrence can amplify vulnerability among the older population. Peterson et al. [3] examined this interplay of food insecurity and functional limitations using national data and found that increasing levels of food insecurity were associated with higher odds of functional limitations in daily activities and IADL among older adults [3]. While the study did not measure QOL directly, these functional impairments are widely recognized as key determinants of diminished health‐related QOL in aging populations, and the finding highlights the synergistic effect of social and physical disadvantage in the aging populations.

While previous research has examined either food insecurity and functional limitations [3], or food insecurity and QOL [5, 10, 17], this study uniquely investigates their combined impact on HRQOL among older adults in an urban setting. Focusing on a racially diverse, aging population in New York City and using validated QOL measures, this study offers a timely and context‐specific contribution to the growing literature on nonclinical determinants of healthy aging. As both food insecurity and ADL limitations are modifiable through community‐based interventions and individual behaviors, studying these dual burdens is essential to improving QOL and reducing disparities among older adults, particularly in urban settings like New York City, where these risks are often concentrated.

This study examines two key nonclinical determinants: food insecurity and limitations in ADLs, and how they intersect to shape health‐related QOL among adults aged 60 and older living in New York City. We hypothesized that individuals experiencing either food insecurity or greater functional limitations would report significantly poorer QOL across physical and mental, domains. By focusing on this unique intersection within an urban aging population, the study contributes new insight into the compounding effects of functional disadvantage on aging outcomes. To our knowledge, few studies have explored these factors together in a localized, city‐level sample, making this research valuable for shaping community‐based programs and public health interventions that support older adults’ independence and well‐being.

## 2. Materials and Methods

### 2.1. Research Design and Recruitment of Participants

This study employs a cross‐sectional, quantitative approach involving 378 participants from New York City. A nonprobability quota sampling strategy was used to recruit participants through Qualtrics‐managed research panels, headquarters in Seattle and Provo, UT, USA [18], between August 2023 and January 2024. Panelists were invited by email or portal notification, and quota controls ensured demographic representation across age, gender, and borough. Eligibility criteria included adults aged 60 and over residing in New York City. Respondents who did not meet age or location criteria or who failed quality checks (e.g., straight‐lining, inconsistent responses) were excluded. This research was approved by the Institutional Review Board at Lehman College (Approval No.: 2023‐0398‐Lehman). All participants gave their consent before proceeding with the online survey. To understand the early stages of aging, we focused on individuals aged 60 and older, a benchmark that help to identify when people might need extra support with their health, mobility, or social connections.

### 2.2. Measures

#### 2.2.1. Demographic Questions

Participants were asked nine demographic questions aiming to identify their age, gender, race/ethnicity, educational background, household income, number of health conditions, and body mass index (BMI). Education, household income, and number of chronic conditions were self‐reported using standard category options with common education and income brackets and standard chronic disease checklists typically used in national health surveys. However, the survey did not use questions adapted from a named validated instrument.

#### 2.2.2. Health‐Related QoL

HRQOL is a way to measure how a person’s physical and mental health contribute positively or negatively to their everyday life. According to Ref. [19], HRQOL is defined as “perceived physical and mental health over time.” The Centers for Disease Control and Prevention created the HRQOL‐4 to measure HRQOL [20]. The HRQOL‐4 consists of four questions that assess individuals’ overall health, the number of days during the past month when their physical or mental health was not good, and the extent to which their physical or mental health prevents them from performing daily activities. The CDC [20] mentioned that to obtain this estimate, responses to Questions 2 and 3 are combined to calculate a summary index of overall unhealthy days in the past month. This instrument was previously used to determine an association between sense of belonging and QOL in New York City [21] and between food security and health‐related QOL [5, 10, 17, 22].

#### 2.2.3. Eastern Cooperative Oncology Group (ECOG)

The ECOG Performance Status scale was used to assess overall functional status and activity limitation. The scale reflects the extent to which health conditions affect an individual’s ability to carry out normal activities, ranging from fully active to severe functional impairment [23]. Participants were asked to select the description they believed most accurately reflected their present mobility and their ability to carry out everyday tasks. This standardized measure, based on an ordinal scale from 0 to 5, was used to determine participants’ scores with 0 = fully active, 1 = limited in physically strenuous activity but active, 2 = ambulatory but unable to carry out work activities, 3 = capable of only limited self‐care, 4 = completely disabled, and 5 = dead. Because this study involved a community survey, no respondents fell into category 5; however, the full‐scale description is retained here for completeness. Higher scores suggest more functional impairment.

#### 2.2.4. Food Insecurity

The Food Security Scale was developed by the U.S. Department of Agriculture [24]. Questions in this study were directed to a group of older adult participants in New York City to determine their relationship to food and whether they experienced food insecurity. Participants were asked about their household food status in the past 12 months using six statements, including “The food we bought didn’t last and we didn’t have money to get more” and “We could not afford to eat balanced meals.” Responses to the statements were yes = 1, no = 0, and I don’t know = 0. The scores were summed, and food security was categorized as high or marginal = 0–1, low = 2–4, and very low = 5–6.

### 2.3. Data Analysis

Participant characteristics were summarized using cell counts and percentages for categorical variables. They included demographics (Manhattan borough, age, gender, race/ethnicity, household income, education, and BMI category), food security status, ECOG performance status, HRQOL‐4 variables, and number of health conditions. The HRQOL‐4 variables included the questions “In general your health is…,” “Number of days in the month your physical health was not good,” “Number of days in the month your mental health was not good,” and “Number of days in the month poor physical or mental health kept you from your usual activities.” Food security status was dichotomized into food secure (high or marginal food security”) or food insecure (very low or low food security). ECOG performance status was categorized as “Fully active,” “Activity limited, can carry out light work,” or “Greater disability or dead.” The “number of days” variables from the HRQOL‐4 were categorized as “0 days,” “1–4 days,” “5–11 days,” or “12+ days.” The number of health conditions was tallied from the yes/no answers of 21 individual health conditions (e.g., Arthritis, yes/no) and was categorized into “0–1,” “2–3,” or “4 or more conditions.”

The responses from the last question on the HRQOL‐4 ask that “In general your health is…” had a 5‐category Likert‐type variable ranging from “Poor” to “Excellent.” It was analyzed as an ordered response variable using the proportional odds regression model for ordinal data. A separate regression model was fitted for each predictor, where the set of predictors included demographics, food security status, and ECOG performance status. The “number of days” variables from the HRQOL‐4 and number of health conditions were analyzed similarly using the proportional odds model. Details of the regression models are presented in supporting tables (available here).

As this study was exploratory in nature, no adjustment for multiple comparisons was planned or implemented. No imputations of missing data were used; only observed data were analyzed. Therefore, the findings should be interpreted with caution. *p* values < 0.05 were considered statistically significant. All analyses were conducted using R, Version 4.3.3 [25]. The proportional odds regression models were fit using the VGAM package (Version 1.1‐10) [26].

## 3. Results

### 3.1. Demographics

A total of 378 participants were included in this study (Table 1). Majority of the participants (47.6%) were between age 60 and 65 years, female (57.9%), and white (48.5%), earned between $35,000 and $74,999 (34.4%), and earned an Associate’s degree. Approximately 64% of the participants were overweight or obese, with two‐thirds having two or more health conditions. Approximately 19% experience food insecurity (low or very low food insecurity) and 56.9% who reported that they were fully active.

**TABLE 1:** Summary of participants: demographics, BMI, health conditions, food security status, and performance status scale (*N* = 378).

Variable	Category	*n* (%)
Age, years (*N* = 358)	60–65	170 (47.5)
	66–75	148 (41.3)
	> 75	40 (11.2)

Gender (*N* = 378)	Female	219 (57.9)
	Male	159 (42.1)

Race/ethnicity (*N* = 377)	Black or African American	59 (15.6)
	Hispanic	104 (27.6)
	white	183 (48.5)
	Other	31 (8.2)

Household income (*N* = 358)	< $15,000	45 (12.6)
	$15,000–$34,999	85 (23.7)
	$35,000–$74,999	123 (34.4)
	≥ $75,000	105 (29.3)

Highest degree earned (*N* = 378)	High school or less	80 (21.2)
	Some college/2 year or associate’s degree	123 (32.5)
	4 year or bachelor’s degree	100 (26.5)
	Graduate degree	75 (19.8)

Body mass index (BMI) (*N* = 378)	Underweight or normal weight (< 25)	136 (36.0)
	Overweight (25–29.9)	125 (33.1)
	Obese (30+)	117 (31.0)

Number of health conditions (*N* = 378)	0‐1	128 (33.9)
	2‐3	145 (38.4)
	4+	105 (27.8)

Food security status (*N* = 356)	Food secure	288 (80.9)
	Food insecure	68 (19.1)

ECOG (*N* = 378)	Fully active	215 (56.9)
	Activity limited; can carry out light work	109 (28.8)
	Greater disability	54 (14.2)

### 3.2. Food Security and HRQOL

Food security status was a significant predictor (*p* = 0.031) of self‐rated general health status (Table 2). Older adults who were food insecure were more likely to rate their health as poor or fair compared to those who were food secure. For example, 10.3% of food‐insecure individuals reported poor health versus 4.2% of food‐secure individuals. Figure 1(a) indicates that, compared to food‐secure older adults, those who experienced food insecurity were more likely to report 12 or more days that their physical health (36.3% vs. 18.1%; *p* < 0.001) and mental (20.9% vs. 9.4%; *p* < 0.001) health were not good. Figure 1 shows that older adults who were food insecure were more likely than those who were food‐secured to report 12 or more days on which their physical or mental health kept them from doing their usual activities (31.1% vs. 9.0%; *p* < 0.001).

**TABLE 2:** Self‐rated general health by sociodemographic conditions, food security status, and functional limitations.

Variable	Category	*n*	General health status					*p* value
			Poor (%)	Fair (%)	Good (%)	Very good (%)	Excellent (%)	
Borough	Bronx	70	2.9	18.6	47.1	27.1	4.3	
	Brooklyn	94	10.6	17.0	39.4	21.3	11.7	0.793
	Manhattan	92	6.5	23.9	34.8	32.6	2.2	0.582
	Queens	92	0.0	14.1	52.2	26.1	7.6	0.411
	Staten Island	29	13.8	20.7	24.1	31.0	10.3	0.869

Age	60–65	170	7.1	17.6	41.8	24.7	8.8	
	66–75	148	4.1	19.6	39.9	29.7	6.8	0.666
	> 75	40	10.0	15.0	47.5	25.0	2.5	0.472

Gender	Female	219	5.0	16.4	45.2	27.9	5.5	
	Male	159	6.9	21.4	37.1	25.8	8.8	0.644

Race	Black or African American	59	3.4	20.3	40.7	30.5	5.1	
	Hispanic	104	4.8	19.2	38.5	26.9	10.6	0.751
	White	183	6.0	18.6	42.6	26.8	6.0	0.739
	Other	31	12.9	9.7	51.6	22.6	3.2	0.470

Income	< 15,000	45	6.7	24.4	42.2	22.2	4.4	
	15,000–34,999	85	9.4	24.7	38.8	21.2	5.9	0.823
	35,000–74,999	123	4.9	17.1	46.3	26.8	4.9	0.316
	≥ 75,000	105	3.8	14.3	36.2	34.3	11.4	0.013

Highest degree	High school or less	80	7.5	25.0	38.8	22.5	6.2	
	Some college/2 years or Assoc. degree	123	8.9	15.4	43.1	29.3	3.3	0.405
	4 years or Bachelor’s degree	100	3.0	24.0	43.0	25.0	5.0	0.518
	Graduate degree	75	2.7	9.3	41.3	30.7	16.0	< 0.001

BMI	Underweight or normal weight (< 25)	136	2.9	11.0	41.9	34.6	9.6	
	Overweight (25–29.9)	125	5.6	20.0	36.8	29.6	8.0	0.063
	Obese (30+)	117	9.4	25.6	47.0	15.4	2.6	< 0.001

Food security status	Food security	288	4.2	16.0	44.1	30.2	5.6	
	Food insecurity	68	10.3	25.0	38.2	13.2	13.2	0.031

ECOG ability to move around	Fully active	215	0.5	7.4	41.9	40.0	10.2	
	Activity limited, can carry out light work	109	8.3	33.9	43.1	12.8	1.8	< 0.001
	Greater disability	54	22.2	31.5	38.9	3.7	3.7	< 0.001

*Note:* The *p* values in the table above are for pairwise comparisons to the first category for the variable. They are derived from the proportional odds regression model for ordinal outcomes, applied separately for each variable. Additional details on the regression models are presented in supporting tables (available here).

FIGURE 1Percentages for number of days with poor health by food security status (a) and functional mobility (b).

**Figure:**
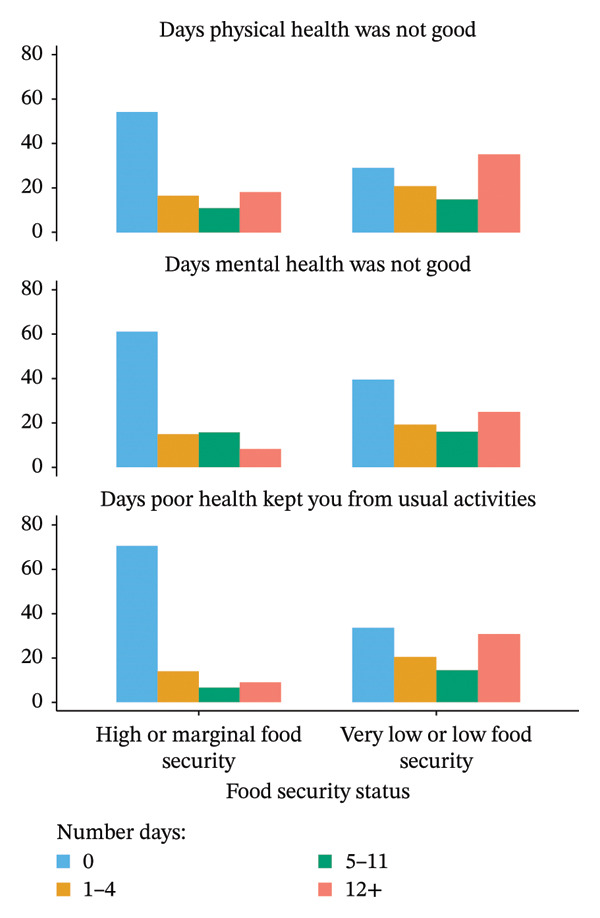
(a)

**Figure:**
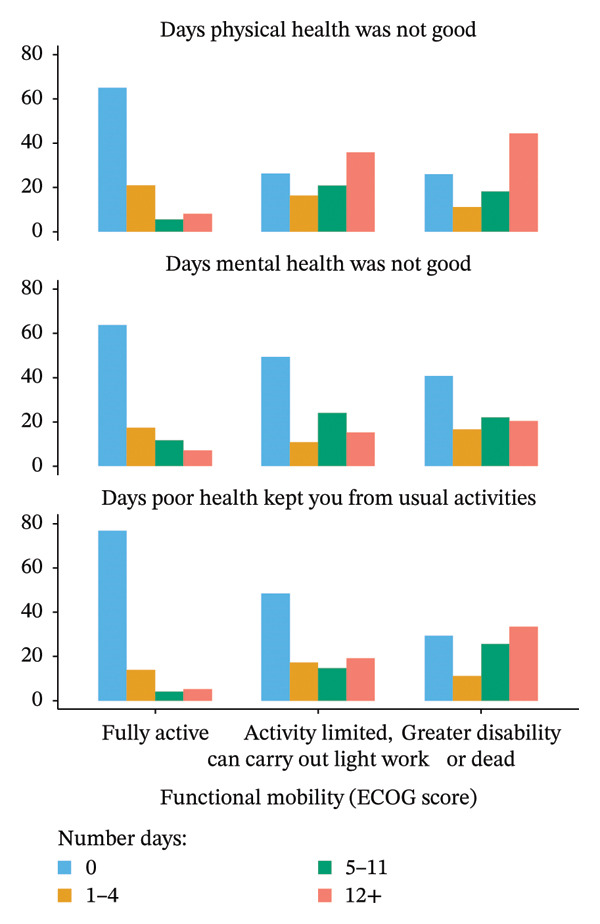
(b)

### 3.3. Functional Mobility and HRQOL

Functional mobility (ECOG scores) was strongly associated with overall health ratings (*p* < 0.001). Only 0.5% of fully active participants reported poor health, in contrast to 22.2% of those with greater disability. Excellent health was reported by 10.2% of fully active individuals compared to only 3.7% of those in the lowest mobility group (Table 2). Functional status and activity limitation, as measured by ECOG score, were also significantly associated with health status across all “number days” domains (*p* < 0.001). Figure 1(b) indicates that participants with more functional limitations or disabilities among older adults were strongly associated with poorer HRQOL. For example, in comparison with fully active older adults, those with activity limitations or disabilities were more likely to report 12 or more days that their physical health (*p* < 0.001) and mental health (*p* < 0.001) were not good. Also, compared to fully active older adults, those who had any activity limitations or disabilities were more likely to report 12 or more days that their physical or mental health kept them from doing their usual activities (*p* < 0.001).

### 3.4. Demographics and HRQOL

Participants’ general health ratings were significantly associated with several demographic and social determinants (Table 2). Educational attainment showed a strong association (*p* = 0.003) with self‐rated health status. Respondents with a graduate degree reported higher ratings of excellent health (16.0%) than those with high school or less (6.2%). Household income was significantly associated with self‐rated health (*p* = 0.013). The group earning ≥ $75,000 had higher proportions reporting excellent health (11.4%) than the group earning < $15,000 (4.4%). BMI category was also a significant predictor of self‐rated health status (*p* < 0.001); “obese” participants (BMI ≥ 30) were more likely to report poor (9.4%) or fair (25.6%) health than those with a “normal” BMI (< 25) (2.9% and 11.0%, respectively).

## 4. Discussion

This study examined factors among older adults at the intersection of food security and functional limitations to determine how these factors are associated with HRQOL. Our findings indicate that food security and greater functional limitations were significantly associated with self‐reported health‐rated quality of life among older adults living in New York City. Similarly, a higher monthly burden of unhealthy days, characterized by suboptimal physical or mental health, was significantly associated with poorer HRQOL. These findings underscore the multifaceted nature of HRQOL in later life and highlight the importance of addressing both social determinants of health and functional limitations when designing interventions aimed at improving well‐being among the aging population.

Our study showed that older adults who are food insecure reported poorer physical, mental, and overall health outcomes. These individuals are also physically and mentally affected for more than 12 days per month, which contributes to their poor perception of their overall health. Chronic food insecurity may promote constant worry and stress about access to adequate foods, which can increase the burden on their physical and psychological health, such as anxiety and depression. This added psychological distress contributes to poor health outcomes. Our findings align with Qian et al. and Aljahdali et al. [17, 27]. For example, Qian et al. [27] conducted a nationally representative study to determine the association between food insecurity and the risk of dementia and memory decline in adults aged 50–80 years. They found that when older adults are food insecure, there is a higher estimated risk of dementia, lower memory scores, and faster memory decline. Similarly, Aljahadali et al. [17] reported that food insecurity was significantly associated with ≥ 16 days of poor physical and mental health. Older adults who experience food insecurity are often affected by inadequate intake of essential nutrients, leading to various health complications such as malnutrition [28–30], muscle weakness, immunocompromised systems, and chronic conditions such as diabetes and hypertension [28, 29]. These cumulative health burdens contribute to poor overall health‐related quality of life [5, 10, 17].

Our results highlight the critical role of functional mobility in shaping the overall health and well‐being of older adults. The strong association between ECOG functional limitation scores and HRQOL suggests that functional limitations challenges are not just a natural part of aging; they may be early warning signs of broader health decline. Just 0.5% of older adults who were fully active described their health as poor, while that number increased significantly to more than 22% among those with greater disability. This striking contrast highlights the significant influence of functional decline on older adults’ perceptions of their overall health. These findings are like previous research showing that difficulties with movement often come before declines in both physical ability and how people perceive their well‐being [31]. Many older adults with limited mobility reported significantly worse physical and mental health, including more days when they felt unwell or were unable to carry out their daily routines, especially if they have a disability. Greater functional disabilities often coexist with pain, greater stress, social isolation, and reduced sense of well‐being [31, 32]. This highlights how closely functional decline is tied to not only physical functioning but also emotional health. These added health burdens also increase older adults’ reliance on health services [31, 32].

This study elucidated the association between food insecurity and functional limitation of activities and their effects on health‐related quality of life. Our current findings have several implications for future practice interventions, public health, and research, concerning our aging population. It is important for practitioners to understand how these factors interact and contribute to HRQOL in older populations in large cities like New York City.

### 4.1. Recommendations

Future studies should aim to determine whether differences exist due to cultural influences and location (city vs. suburban vs. rural). Assessments of cultural and ethnic backgrounds, practices, and values can shape perceptions about food and illness, which can influence health outcomes. Future research should explore the influences of location and culture on our aging population. We recommend that future researchers explore improving access to quality healthcare, promoting healthy lifestyle behaviors, and implementing culturally sensitive interventions tailored to our aging population. Our study did not include access to healthcare or whether older adults were receiving food assistance from programs like Supporting Nutrition Assistance Program (SNAP). Therefore, future studies should incorporate these variables as they will give more insights into the multifaceted nature of food insecurity and its intersection with health. Including such dimensions would allow for a more comprehensive understanding of the vulnerabilities experienced by older populations and inform more targeted policy interventions.

### 4.2. Strengths and Limitations

One major strength of this study is our assessment of the variables (food insecurity and functional status and activity limitations) simultaneously on the health‐related quality of life among older adults. The results showed that while both variables showed a statistically significant impact on HRQOL, a stronger association exists between individuals who experience activity limitation or greater disability (*p* < 0.001) than those who experience food insecurity (*p* = 0.031). A possible explanation is that when individuals have limited mobility, it can impact their independence, including their ability to secure healthy, nutritious, and safe foods. This can have a greater negative impact on their health than if they are fully active and they suddenly become inactive. Fully active individuals are more likely to move around and interact with others and are more independent, therefore, they feel a higher sense of purpose and view their health more positively.

Our study has several limitations. The sample size was smaller than we anticipated and lacked adequate racial distributions; however, the demographics that responded aligned with the national demographics of the United States where majority whites were most likely to respond. Additionally, because participant recruitment was conducted via a third‐party panel provider (Qualtrics), we did not have access to the total number of individuals invited or exposed to the survey, which limits our ability to present this information. Recall bias may have limited what older adult participants remembered regarding their food security status. Thirdly, technology bias may have skewed toward participants who owned a computer versus those who did not. It is also important to note that this digital survey likely did not reach seniors who struggle with technology, have fewer resources, or are too frail to go online due to nonprobability online sampling. As a result, the experiences of the most vulnerable and marginalized older adults in NYC may be missing from this data. Fourthly, we did not conduct multiple regression analyses; therefore, the results of this study must be interpreted with caution. Finally, because this was strictly an exploratory study, the statistical tests were not adjusted for multiple testing, and therefore, the *p* values must also be interpreted with caution. For these reasons as well as a relatively small sample size, the findings of this study cannot be generalized beyond the study population.

## 5. Conclusion

Food insecurity and functional limitations are independently associated with health‐related quality of life among older adults. These mechanisms can contribute to suboptimal QOL and increased need for health services, especially when they impact physical and psychological health. These findings highlight the importance of integrated interventions that address food insecurity and resources that support physical functioning to improve the overall health of older adults.

## Author Contributions

Conceptualization, Adjara Stewart, Christine Rodriguez, Destiny Lopez, Nelia Jose, Mya Encarnacion, and Collette M. Brown; methodology, Collette M. Brown; validation, Collette M. Brown and Elgloria A. Harrison; formal analysis, Apeksha Mewani and Collette M. Brown; writing, Adjara Stewart, Christine Rodriguez, Destiny Lopez, Nelia Jose, Mya Encarnacion, Collette M. Brown, Apeksha Mewani, Sheila Hankin, and Elgloria A. Harrison; and writing–review and editing, Collette M. Brown, and Apeksha Mewani.

## Funding

This research has no external funding.

## Disclosure

All authors have read and agreed to the published version of the manuscript.”

## Conflicts of Interest

The authors declare no conflicts of interest.

## Supporting Information

Additional supporting information can be found online in the Supporting Information section.

## Supporting information


**Supporting Information** Supporting Table 1: the results of the proportional odds of examining the relationship between borough and general health status. There is no statistical evidence that borough of residence is associated with general health status. Supporting Table 1: proportional odds analysis of general health status by borough. Supporting Table 2 reports the results the proportional odds of examining the relationship between age and general health status. Age was not significantly associated with general health status. Supporting Table 2: proportional odds analysis of general health status by borough by age. Supporting Table 3: the results the proportional odds of examining the relationship between gender and general health status. Gender was not significantly associated with general health status. Supporting Table 3: proportional odds analysis of general health status by gender. Supporting Table 4 reports the results the proportional odds of examining the relationship between race and general health status. Race was not significantly associated with general health status. Supporting Table 4: proportional odds analysis of general health status by race. Supporting Table 5 reports the results the proportional odds of examining the relationship between household income and general health status. Income was significantly associated with general health status (X2 = 12.65; df = 3; *p* = 0.005). Individuals with household income ≥ $75,000 had significantly higher odds of reporting better health compared to those earning < $15,000 (β = −0.816, *p* = 0.013), while lower income categories were not significantly different from those who earned < $15,000. Supporting Table 5: proportional odds analysis of general health status by income. Supporting Table 6: the results the proportional odds of examining the relationship between education and general health status. Education was significantly associated with general health status (X2 = 13.61; df = 3; *p* = 0.003). Individuals who earned a graduate degree had significantly higher odds of reporting better health compared to those who completed high school or less (β = −1.010, *p* = 0.0001), while those who had some college degree or earned a bachelor’s degree were not significantly different in reporting health status from those had high school or less education. Supporting Table 6: proportional odds analysis of general health status by the highest degree. Supporting Table 7 reports the results the proportional odds of examining the relationship between BMI and general health status. The BMI was significantly associated with general health status (X2 = 24.29; df = 2; *p* = 0.001). Individuals who were obese had significantly higher odds of reporting poorer general health compared to those who were underweight (β = 1.168, *p* < 0.0001). Supporting Table 7: proportional odds analysis of general health status by BMI category. Supporting Table 8: the results the proportional odds of examining the relationship between food security status and general health status. Food security status was significantly associated with general health status (X2 = 4.65; df = 1; *p* = 0.031). Individuals who experienced low or very low food insecurity had significantly higher odds of reporting poorer general health compared to those who experienced marginal of high food security (β = 0.536, *p* < 0.031). Supporting Table 8: proportional odds analysis of general health status by food security status. Supporting Table 9 reports the results the proportional odds of examining the relationship between Activity Limitation and general health status. Activity limitation was significantly associated with general health status (X2 = 92.49; df = 2; *p* < 0.001). Compared to fully active individuals, those with activity limitations had nearly seven times the odds of reporting poorer health (β = 1.932, *p* < 0.001), while those with greater disability had over thirteen times the odds (β = 2.614, *p* < 0.001), demonstrating a pronounced gradient between functional impairment and health status. Supporting Table 9: proportional odds analysis of general health status by ECOG ability to move around. Supporting Table 10 shows the association between food security status and days of poor physical health. Food insecurity was significantly associated with more days of poor physical health. Supporting Table 10: proportional odds analysis of days physical health was not good by food security status. Supporting Table 11: the association between functional status (ECOG) and days of poor physical health. Functional limitations were significantly associated with more days of poor physical health. Supporting Table 11: proportional odds analysis of days physical health was not good by ECOG ability to move around. Supporting Table 12: the association between food security status and days of poor mental health. Food insecurity was significantly associated with more days of poor mental health. Supporting Table 12: proportional odds analysis of days mental health was not good by food security status. Supporting Table 13: The association between functional status (ECOG) and days of poor mental health. Functional limitations were significantly associated with more days of poor mental health Supporting Table 13: proportional odds analysis of days mental health was not good by ECOG ability to move around. Supporting Table 14 shows the association between food security status and days poor health limited usual activities. Food insecurity was significantly associated with more days of activity limitation. Supporting Table 14: proportional odds analysis of days poor health kept you from usual activities by food security status. Supporting Table 15 shows the association between functional status (ECOG) and days poor health limited usual activities. Functional limitations were significantly associated with greater activity limitation. Supporting Table 15: proportional odds analysis of days poor health kept you from usual activities by ECOG ability to move around.

## Data Availability

The raw data supporting the conclusions of this article will be made available by the authors on request.
