# Supplementary material for: Exploring Food Security, Functional Limitations, and Quality of Life Among Adults 60 Years and Older in New York City: A Cross‐Sectional Study
Source: J Aging Res. 2026 May 25;2026:1291602. doi: 10.1155/jare/1291602 (PMC13199911; doi:10.1155/jare/1291602)
Supplement: Supplementary file 1 — Supporting Information Supporting Table 1: the results of the proportional odds of examining the relationship between borough and general health status. There is no statistical evidence that borough of residence is associated with general health status. Supporting Table 1: proportional odds analysis of general health status by borough. Supporting Table 2 reports the results the proportional odds of examining the relationship between age and general health status. Age was not significantly associated with general health status. Supporting Table 2: proportional odds analysis of general health status by borough by age. Supporting Table 3: the results the proportional odds of examining the relationship between gender and general health status. Gender was not significantly associated with general health status. Supporting Table 3: proportional odds analysis of general health status by gender. Supporting Table 4 reports the results the proportional odds of examining the relationship between race and general health status. Race was not significantly associated with general health status. Supporting Table 4: proportional odds analysis of general health status by race. Supporting Table 5 reports the results the proportional odds of examining the relationship between household income and general health status. Income was significantly associated with general health status (X2 = 12.65; df = 3; p = 0.005). Individuals with household income ≥ $75,000 had significantly higher odds of reporting better health compared to those earning < $15,000 (β = −0.816, p = 0.013), while lower income categories were not significantly different from those who earned < $15,000. Supporting Table 5: proportional odds analysis of general health status by income. Supporting Table 6: the results the proportional odds of examining the relationship between education and general health status. Education was significantly associated with general health status (X2 = 13.61; df = 3; p = 0.003). Individuals w [file JARE-2026-1291602-s001.docx]

Supplemental Table 1: Proportional Odds Analysis of GENERAL HEALTH STATUS

by BOROUGH

Regression Coefficients from Proportional Odds Model (cumulative logit model)

Term Estimate Std. Error z value Pr(>|z|)

------------------------------------------------------------

(Intercept):1 -2.791 0.295 -9.457 <0.001

(Intercept):2 -1.131 0.231 -4.905 <0.001

(Intercept):3 0.672 0.225 2.986 0.003

(Intercept):4 2.608 0.283 9.207 <0.001

boroBrooklyn 0.076 0.288 0.263 0.793

boroManhattan 0.159 0.290 0.550 0.582

boroQueens -0.238 0.290 -0.822 0.411

boroStaten Island 0.066 0.403 0.164 0.869

------------------------------------------------------------

Reference category for BOROUGH: Bronx.

Wald test of BOROUGH

Chi-Sq df p-value

---------------------

2.45 4 0.654

---------------------

Supplemental Table 2: Proportional Odds Analysis of GENERAL HEALTH STATUS

by AGE

Regression Coefficients from Proportional Odds Model (cumulative logit model)

Term Estimate Std. Error z value Pr(>|z|)

--------------------------------------------------------

(Intercept):1 -2.718 0.242 -11.226 <0.001

(Intercept):2 -1.127 0.159 -7.071 <0.001

(Intercept):3 0.671 0.151 4.447 <0.001

(Intercept):4 2.561 0.228 11.239 <0.001

age266-75 -0.089 0.205 -0.432 0.666

age2> 75 0.230 0.321 0.719 0.472

--------------------------------------------------------

Reference category for AGE: 60-65.

Wald test of AGE

Chi-Sq df p-value

---------------------

0.97 2 0.615

---------------------

Supplemental Table 3: Proportional Odds Analysis of GENERAL HEALTH STATUS

by GENDER

Regression Coefficients from Proportional Odds Model (cumulative logit model)

Term Estimate Std. Error z value Pr(>|z|)

--------------------------------------------------------

(Intercept):1 -2.821 0.235 -12.023 <0.001

(Intercept):2 -1.170 0.145 -8.073 <0.001

(Intercept):3 0.635 0.134 4.722 <0.001

(Intercept):4 2.570 0.218 11.812 <0.001

genderMale 0.088 0.190 0.462 0.644

--------------------------------------------------------

Reference category for GENDER: Female.

Wald test of GENDER

Chi-Sq df p-value

---------------------

0.21 1 0.644

---------------------

Supplemental Table 4: Proportional Odds Analysis of GENERAL HEALTH STATUS

by RACE

Regression Coefficients from Proportional Odds Model (cumulative logit model)

Term Estimate Std. Error z value Pr(>|z|)

--------------------------------------------------------

(Intercept):1 -2.827 0.311 -9.102 <0.001

(Intercept):2 -1.191 0.250 -4.764 <0.001

(Intercept):3 0.622 0.244 2.553 0.011

(Intercept):4 2.564 0.298 8.614 <0.001

race2Hispanic -0.094 0.298 -0.317 0.751

race2White 0.091 0.273 0.333 0.739

race2other 0.293 0.405 0.722 0.470

--------------------------------------------------------

Reference category for RACE: Black or African American.

Wald test of RACE

Chi-Sq df p-value

---------------------

1.32 3 0.723

---------------------

Supplemental Table 5: Proportional Odds Analysis of GENERAL HEALTH STATUS

by INCOME

Regression Coefficients from Proportional Odds Model (cumulative logit model)

Term Estimate Std. Error z value Pr(>|z|)

--------------------------------------------------------------------

(Intercept):1 -2.496 0.335 -7.445 <0.001

(Intercept):2 -0.805 0.279 -2.881 0.004

(Intercept):3 1.008 0.282 3.581 <0.001

(Intercept):4 2.972 0.336 8.857 <0.001

HH_income215,000 - 34,999 0.075 0.337 0.224 0.823

HH_income235,000 - 74,999 -0.319 0.319 -1.003 0.316

HH_income2>= 75,000 -0.816 0.328 -2.487 0.013

--------------------------------------------------------------------

Reference category for INCOME: < 15,000.

Wald test of INCOME

Chi-Sq df p-value

---------------------

12.65 3 0.005

---------------------

Supplemental Table 6: Proportional Odds Analysis of GENERAL HEALTH STATUS

by HIGHEST DEGREE

Regression Coefficients from Proportional Odds Model (cumulative logit model)

Term Estimate Std. Error z value Pr(>|z|)

---------------------------------------------------------------------------------------------

(Intercept):1 -2.514 0.280 -8.977 <0.001

(Intercept):2 -0.845 0.214 -3.950 <0.001

(Intercept):3 1.002 0.216 4.640 <0.001

(Intercept):4 2.975 0.281 10.598 <0.001

highest_degree2some college/2 year or assoc degree -0.219 0.263 -0.833 0.405

highest_degree24 year or Bachelors degree -0.177 0.274 -0.646 0.518

highest_degree2Graduate degree -1.010 0.298 -3.385 <0.001

---------------------------------------------------------------------------------------------

Reference category for HIGHEST DEGREE: high school or less.

Wald test of HIGHEST DEGREE

Chi-Sq df p-value

---------------------

13.61 3 0.003

---------------------

Supplemental Table 7: Proportional Odds Analysis of GENERAL HEALTH STATUS

by BMI CATEGORY

Regression Coefficients from Proportional Odds Model (cumulative logit model)

Term Estimate Std. Error z value Pr(>|z|)

----------------------------------------------------------------------------

(Intercept):1 -3.391 0.268 -12.641 <0.001

(Intercept):2 -1.699 0.188 -9.049 <0.001

(Intercept):3 0.203 0.163 1.245 0.213

(Intercept):4 2.196 0.230 9.536 <0.001

BMI_category2overweight (25-29.9) 0.425 0.228 1.860 0.063

BMI_category2obese (30+) 1.168 0.238 4.899 <0.001

----------------------------------------------------------------------------

Reference category for BMI CATEGORY: underweight or normal weight (<25).

Wald test of BMI CATEGORY

Chi-Sq df p-value

---------------------

24.29 2 <0.001

---------------------

Supplemental Table 8: Proportional Odds Analysis of GENERAL HEALTH STATUS

by FOOD SECURITY STATUS

Regression Coefficients from Proportional Odds Model (cumulative logit model)

Term Estimate Std. Error z value Pr(>|z|)

---------------------------------------------------------------------------------------------

(Intercept):1 -2.996 0.244 -12.289 <0.001

(Intercept):2 -1.312 0.138 -9.519 <0.001

(Intercept):3 0.582 0.120 4.846 <0.001

(Intercept):4 2.506 0.211 11.853 <0.001

food_security_status2Very low or low food security 0.536 0.249 2.155 0.031

---------------------------------------------------------------------------------------------

Reference category for FOOD SECURITY STATUS: High or marginal food security.

Wald test of FOOD SECURITY STATUS

Chi-Sq df p-value

---------------------

4.65 1 0.031

---------------------

Supplemental Table 9: Proportional Odds Analysis of GENERAL HEALTH STATUS

by ECOG ABILITY TO MOVE AROUND

Regression Coefficients from Proportional Odds Model (cumulative logit model)

Term Estimate Std. Error z value Pr(>|z|)

----------------------------------------------------------------------------------------------------------------------

(Intercept):1 -4.207 0.284 -14.815 <0.001

(Intercept):2 -2.325 0.197 -11.819 <0.001

(Intercept):3 -0.021 0.134 -0.155 0.877

(Intercept):4 2.100 0.210 9.980 <0.001

ECOG_your_ability_to_move_around2Activity limited, can carry out light work 1.932 0.246 7.852 <0.001

ECOG_your_ability_to_move_around2Greater disabilty or dead 2.614 0.315 8.288 <0.001

----------------------------------------------------------------------------------------------------------------------

Reference category for ECOG ABILITY TO MOVE AROUND: Fully Active.

Wald test of ECOG ABILITY TO MOVE AROUND

Chi-Sq df p-value

---------------------

92.49 2 <0.001

---------------------

Supplemental Table 10: Proportional Odds Analysis of DAYS PHYSICAL HEALTH WAS NOT GOOD

by FOOD SECURITY STATUS

Regression Coefficients from Proportional Odds Model (cumulative logit model)

Term Estimate Std. Error z value Pr(>|z|)

---------------------------------------------------------------------------------------------

(Intercept):1 0.156 0.117 1.333 0.183

(Intercept):2 0.906 0.127 7.150 <0.001

(Intercept):3 1.510 0.144 10.462 <0.001

food_security_status2Very low or low food security -0.948 0.249 -3.811 <0.001

---------------------------------------------------------------------------------------------

Reference category for FOOD SECURITY STATUS: High or marginal food security.

Wald test of FOOD SECURITY STATUS

Chi-Sq df p-value

---------------------

14.53 1 <0.001

---------------------

Supplemental Table 11: Proportional Odds Analysis of DAYS PHYSICAL HEALTH WAS NOT GOOD

by ECOG ABILITY TO MOVE AROUND

Regression Coefficients from Proportional Odds Model (cumulative logit model)

Term Estimate Std. Error z value Pr(>|z|)

----------------------------------------------------------------------------------------------------------------------

(Intercept):1 0.667 0.142 4.685 <0.001

(Intercept):2 1.597 0.163 9.770 <0.001

(Intercept):3 2.332 0.186 12.559 <0.001

ECOG_your_ability_to_move_around2Activity limited, can carry out light work -1.768 0.231 -7.647 <0.001

ECOG_your_ability_to_move_around2Greater disabilty or dead -2.025 0.294 -6.885 <0.001

----------------------------------------------------------------------------------------------------------------------

Reference category for ECOG ABILITY TO MOVE AROUND: Fully Active.

Wald test of ECOG ABILITY TO MOVE AROUND

Chi-Sq df p-value

---------------------

78.31 2 <0.001

---------------------

Supplemental Table 12: Proportional Odds Analysis of DAYS MENTAL HEALTH WAS NOT GOOD

by FOOD SECURITY STATUS

Regression Coefficients from Proportional Odds Model (cumulative logit model)

Term Estimate Std. Error z value Pr(>|z|)

---------------------------------------------------------------------------------------------

(Intercept):1 0.460 0.120 3.831 <0.001

(Intercept):2 1.177 0.134 8.767 <0.001

(Intercept):3 2.261 0.181 12.494 <0.001

food_security_status2Very low or low food security -0.930 0.251 -3.712 <0.001

---------------------------------------------------------------------------------------------

Reference category for FOOD SECURITY STATUS: High or marginal food security.

Wald test of FOOD SECURITY STATUS

Chi-Sq df p-value

---------------------

13.78 1 <0.001

---------------------

Supplemental Table 13: Proportional Odds Analysis of DAYS MENTAL HEALTH WAS NOT GOOD

by ECOG ABILITY TO MOVE AROUND

Regression Coefficients from Proportional Odds Model (cumulative logit model)

Term Estimate Std. Error z value Pr(>|z|)

----------------------------------------------------------------------------------------------------------------------

(Intercept):1 0.604 0.141 4.292 <0.001

(Intercept):2 1.306 0.154 8.505 <0.001

(Intercept):3 2.442 0.196 12.491 <0.001

ECOG_your_ability_to_move_around2Activity limited, can carry out light work -0.719 0.226 -3.184 0.001

ECOG_your_ability_to_move_around2Greater disabilty or dead -1.010 0.285 -3.544 <0.001

----------------------------------------------------------------------------------------------------------------------

Reference category for ECOG ABILITY TO MOVE AROUND: Fully Active.

Wald test of ECOG ABILITY TO MOVE AROUND

Chi-Sq df p-value

---------------------

17.38 2 <0.001

---------------------

Supplemental Table 14: Proportional Odds Analysis of DAYS POOR HEALTH KEPT YOU FROM USUAL ACTIVITIES

by FOOD SECURITY STATUS

Regression Coefficients from Proportional Odds Model (cumulative logit model)

Term Estimate Std. Error z value Pr(>|z|)

---------------------------------------------------------------------------------------------

(Intercept):1 0.869 0.129 6.760 <0.001

(Intercept):2 1.693 0.154 10.997 <0.001

(Intercept):3 2.319 0.183 12.659 <0.001

food_security_status2Very low or low food security -1.525 0.258 -5.901 <0.001

---------------------------------------------------------------------------------------------

Reference category for FOOD SECURITY STATUS: High or marginal food security.

Wald test of FOOD SECURITY STATUS

Chi-Sq df p-value

---------------------

34.82 1 <0.001

---------------------

Supplemental Table 15: Proportional Odds Analysis of DAYS POOR HEALTH KEPT YOU FROM USUAL ACTIVITIES

by ECOG ABILITY TO MOVE AROUND

Regression Coefficients from Proportional Odds Model (cumulative logit model)

Term Estimate Std. Error z value Pr(>|z|)

----------------------------------------------------------------------------------------------------------------------

(Intercept):1 1.221 0.162 7.551 <0.001

(Intercept):2 2.040 0.185 11.053 <0.001

(Intercept):3 2.851 0.215 13.239 <0.001

ECOG_your_ability_to_move_around2Activity limited, can carry out light work -1.326 0.242 -5.483 <0.001

ECOG_your_ability_to_move_around2Greater disabilty or dead -2.214 0.302 -7.334 <0.001

----------------------------------------------------------------------------------------------------------------------

Reference category for ECOG ABILITY TO MOVE AROUND: Fully Active.

Wald test of ECOG ABILITY TO MOVE AROUND

Chi-Sq df p-value

---------------------

61.98 2 <0.001

---------------------
